# Predicting habitat suitability and niche dynamics of *Dactylorhiza hatagirea* and *Rheum webbianum* in the Himalaya under projected climate change

**DOI:** 10.1038/s41598-022-16837-5

**Published:** 2022-08-01

**Authors:** Ishfaq Ahmad Wani, Sajid Khan, Susheel Verma, Fahad A. Al-Misned, Hesham M. Shafik, Hamed A. El-Serehy

**Affiliations:** 1 Conservation and Molecular Biology Laboratory, Department of Botany, Baba Ghulam Shah Badshah University, Rajouri, 185234 India; 2 Department of Botany, University of Jammu, Jammu, 180006 India; 3 Department of Zoology, College of Science, King Saud University, Riyadh, 11451 Saudi Arabia; 4 Hungarian Academy of Sciences, Limnoecology Research Group, University of Pannonia, Gyetem, Hungary

**Keywords:** Climate sciences, Environmental sciences

## Abstract

In the era of anthropocene, global warming tends to alter the distribution range of the plant species. Highly fragile to such changes are the species that are endemic, inhabit higher elevations and show narrow distribution ranges. Predicting and plotting the appropriate suitable habitats and keeping knowledge of how climate change will affect future distribution become imperative for designing effective conservation strategies. In the current study we have used BIOMOD ensemble forecasting to study the current and predict the future potential distribution of *Dactylorhiza hatagirea* and *Rheum webbianum* and describe their niche dynamics in Himalayan biodiversity hotspots under climate change scenarios using *ecospat R* package. Results reveal sufficient internal evaluation metrics with area under curve (AUC) and true skill statistic (TSS) values greater than 0.8 i.e. 0.93 and 0.98 and 0.82 and 0.90 for *D. hatageria* and *R. webbianum* respectively, which signifies robustness of the model. Among different bioclimatic variables, bio_1, bio_3, bio_8, bio_14 and bio_15 were the most influential, showing greater impact on the potential distribution of these plant species. Range change analysis showed that both the studied species will show significant contraction of their suitable habitats under future climatic scenarios. Representative Concentration Pathway (RCP) 8.5 for the year 2070, indicate that the suitable habitats could be reduced by about 51.41% and 70.57% for *D. hatagirea* and *R. webbianum* respectively. The results of the niche comparisons between the current and future climatic scenarios showed moderate level of niche overlap for all the pairs with *D. hatageria* showing 61% overlap for current vs. RCP4.5 2050 and *R. webbianum* reflects 68% overlap for current vs. RCP4.5 2050. Furthermore, the PCA analysis revealed that climatic conditions for both the species vary significantly between current and future scenarios. The similarity and equivalence test showed that the niche between present and future climate change scenarios is comparable but not identical. From the current study we concluded that the influence of climate change on the habitat distribution of these plant species in the Himalayan biodiversity hotspots can be considered very severe. Drastic reduction in overall habitat suitability poses a high risk of species extinction and thereby threatens to alter the functions and services of these fragile ecosystems. Present results can be used by conservationists for mitigating the biodiversity decline and exploring undocumented populations on one hand and by policymakers in implementing the policy of conservation of species by launching species recovery programmes in future on the other. The outcomes of this study can contribute substantially to understand the consequences of climate change in the Himalayan biodiversity hotspots.

## Introduction

The Himalaya are the highest and the youngest mountainous landscapes of the world and are recognized as the global biodiversity hotspots^1^. They represent a rich repository of biodiversity due to varied ecological, bio-geographical and evolutionary factors that favour high endemic biodiversity^2^. Greater diversity of altitude, rainfall and soil conditions generates a variety of eco-regions, harbouring about 10,000 plant species among which 3160 are endemic^3^. Climate change effects are experienced by all types of ecosystems and species, but the Himalayan ecosystems are highly vulnerable to natural hazards, that leads to raising concerns about climate change impacts on the biodiversity of these regions^4–6^. According to many model-based estimates of climate change impacts on plant diversity, mountain ecosystems may be among the most susceptible of all terrestrial ecosystems because their floristic composition is generally limited by low temperatures^7–9^. Besides climate change is expected to shift the plant species towards increasing dominance of warm-adapted species and loss of cold adapted species (thermophilization)^10^. The climate induced warming in the Himalaya is experiencing faster rates (0.06 °C/year) than predicted for the other regions of the world^11–13^, raising the likelihood of species extinction in these vulnerable regions. Analyses of temperature trends have shown that temperature increases are greater at the higher altitudes than in the lowlands^14^. Studies suggest that a 0.3 °C rise in global mean temperature per decade will result in potential rise of the global mean surface temperature of about 1–3.5 °C by 2100^15^. Each 1 °C of temperature change moves ecological zones on Earth by about 160 km, i.e., if the climate warms by 4 °C over the next century, plant species have to migrate some 500 m higher in altitude to find a suitable habitat with appropriate climatic conditions^16^. Evidence of the sensitivity of alpine habitats is provided by shifts in the altitudinal-range margins of plant species and bioclimatic zones in the past 50 years, with upward displacement of 120–340 m for tree and woody shrub species^17^ and upward migration of alpine and nival plant species at a rate of 8–10 m per decade^18,19^. Plant species within alpine habitats are at greater risk than lower altitudes for habitat loss. Reports reveal that 36–55% of alpine species, 31–51% of subalpine species, and 19–46% of alpine species will lose more than 80% of their suitable habitat by 2070–2100 due to climate change^20^. On account of global warming and changes in precipitation pattern, appropriate habitats for several high-altitude plant species could be severely altered or vanished by the end of twenty-first century^21–23^. As a result, it has been proposed that the application of distribution models to determine the extent of species occurrence should be the central concept of different biodiversity assessment and conservation schemes^24,25^.

Data analysis and statistical tools, such as species distribution models (SDMs) are significant tools that integrate presence and pseudo-absence data with abiotic data^26,27^. To achieve ecological and biological management of different species, SDMs hold a prime repute in predicting their geographic distribution^5^. Differences among various SDM algorithms make it challenging to choose the best model^26–28^. To overcome this issue, the ensemble modelling (ENM) approach using BIOMOD serves as a suitable platform to examine the distribution range of species and how it could alter as a result of climate change^29,30^. BIOMOD combines together various statistical and machine learning methods to improve habitat suitability estimation^31–33^. Statistical methods, such as multivariate adaptive regression spline (MARS), the flexible discriminant analysis (FDA), the generalized linear model (GLM) and machine-learning methods, such as classification tree analysis (CTA), maximum entropy (MaxEnt), the generalized boost model (GBM), artificial neural network (ANN), random forest (RF), examines the different linear associations between environmental layers and species distributions. Detailed description of the algorithms is provided in Table 1. In order to evaluate the impact of climate change on species distribution, Representative Concentration Pathways (RCPs) which determine the probable emission of greenhouse gases and air pollutants in the atmosphere must be considered for different time scenarios (RCP 4.5 and 8.5 for 2050 and 2070) to provide trajectories for climate change^34–39^. The main aim of developing the RCPs is to generate the information related to possible development of trajectories for the main forcing agents that are the primer drivers of climate change, in consistent with current scenario. This allows subsequent analysis by both Integrated Assessment Models (IAMs) and Climate models (CMs). The time series of RCPs that represent future concentrations and emissions of greenhouse gases and air pollutants and land-use change will be used by climate modellers to undergo new experiments related to climate change and produce new climate scenarios^40^.

**Table 1:** Overview of the algorithms of BIOMOD modelling technique used for determining the habitat suitability.

Models	Descriptions	Category	References
Generalized Additive Model (GAM)	It is based on the relation between random and systematic component	Statistical regression	^41^
Multivariate Adaptive Regression Spline (MARS)	It generates a number of linear regression models spanning a wide range of predictor values	Statistical regression	^42^
Flexible Discriminant Analysis (FDA)	It is a classification approach that uses a combination of linear regression models	Statistical regression	^32^
Generalized Boosting Model (GBM)	It is built using a combination of decision tree algorithms and boosting techniques	Machine learning	^43,44^
Maximum Entropy (MAXENT)	It is used to make prediction from incomplete knowledge	Machine learning	^45,46^
Random Forest (RF)	It forms a set of decision trees using bootstrap aggregation	Machine learning	^47,48^
Classification of Tree Analysis (CTA)	It is a supervised non-parametric statistical classification approach that is based on binary recursive partitioning techniques	Machine learning	^44,49^
Artificial Neural Network (ANN)	It is based on non-linear mapping structures inspired on the biological system of the brain	Machine learning	^50,51^
Surface Range Envelope (SRE)	It is a technique that is based on environmental conditions of occurrence points	Profile	^52,53^

In the current study, we have tried to map the present and future distribution of two threatened medicinal plants (*D. hatagirea* and *R. webbianum*) across the entire range of Himalayan biodiversity hotspots by utilising the BIOMOD Ensemble modelling approach. Their niche dynamics was performed using *Ecospat* package in R software^54^. Our target species are the typical alpine to sub-alpine species which are endemic to Himalayan mountains. Until now, distribution modelling approaches for *D. hatagirea* have been performed at local scale which is focused on the current distribution pattern and is based on single algorithms i.e., MaxEnt^55–57^. For *Rheum webbianum* no such studies have been conducted so far. In particular, the aim of this study is to assess the potential habitat distribution of these species under current and future climatic conditions. This study will also help us to delineate the range contraction or expansion of suitable habitats and to assess the impact of potential climatic change on their niche overlap between current and future climatic scenarios. Keeping in view the threat status of these plant species (Table 2), current study could be more appropriate as far as conservation plans for these Himalayan endemic species are concerned. These studies will not only facilitate the researchers to explore their new locations but will also be helpful to different stakeholders, NGOs and conservation biologists to restore their degraded habitats.

**Table 2:** Threat status of the plant species under study.

Plant species	Vernacular same	Threat status	References
*Dactylorhiza hatagirea*	Himalayan Marsh Orchid	Critically endangered	^58,59^
*Rheum webbianum*	Himalayan Rhubarb	Endangered	^60^

## Results

### Habitat suitability

#### Model evaluation

The final ensemble models produced had an AUC value equal to 0.93 and 0.98 and TSS values equal to 0.82 and 0.90 for *Dactylorhiza hatageria* and *Rheum webbianum*, respectively (Table 3). Both of these scores suggest that the final models were robust in predicting the distribution of the studied species.

**Table 3:** Final AUC (ROC) values of *Dactylorhiza hatagirea* and *Rheum webbianum.*

Species	Final Ensemble values				
		Testing data	Cut off	Sensitivity	Specificity
*D. hatagirea*	AUC	0.93	663.7	91.1	90.9
	TSS	0.82	682.6	91.6	90.2
*R. webbianum*	AUC	0.98	482.2	89.9	88.43
	TSS	0.90	471.1	89.7	87.7

**Figure 1:**
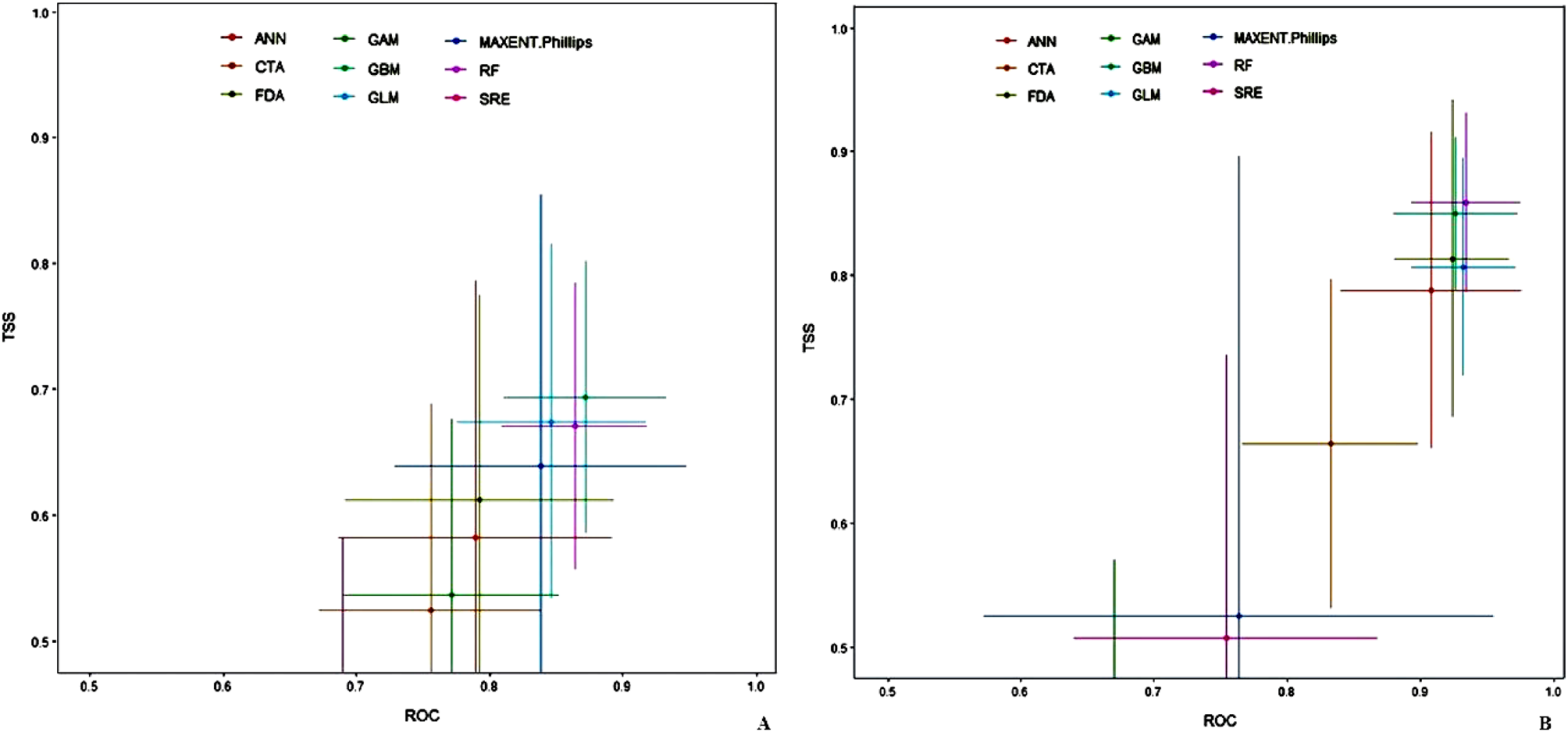
Mean model evaluation scores by algorithms according to two different evaluation metrics, Receivers Operating Characteristic Curve (ROC) and True Skill Statistics (TSS) for (**A**) *Dactylorhiza hatageria* (**B**) *Rheum webbianum.*

When compared at the individual algorithm level, the predictive accuracy was excellent, but varied with Generalised Boosted Models (GBM), Random Forest (RF), Generalised Linear Model (GLM) performing fairly well, followed by Maxent Phillips (MaxEnt), Flexible Discriminant Analysis (FDA) and Artifical Neural Network (ANN), while as the algorithms such as Generalized Additive Model (GAM), Classification of Tree Analysis (CTA) and Surface Range Envelope (SRE) showed lowest accuracy when compared to rest of the algorithms used for *Dactylorhiza hatageria* (Fig. 1A). Similarly, in case of *Rheum webbianum,* the algorithms GBM, RF, GLM and MaxEnt achieved the highest accuracy, followed by FDA, while as CTA, SRE, ANN and GAM performed relatively lowest when compared to rest of the algorithms (Fig. 1B).

#### Variable importance

The importance of selected predictor variables showed greater variation across different algorithms. More specifically in case of *Dactylorhiza hatageria* the most influential variables were bio_08 (Mean Temperature of Wettest Quarter) with importance scores ranging from 0.26 and 0.84 followed by bio_01 (Annual Mean Temperature) with importance scores ranging from 0.16 to 0.59 and bio_03 (Isothermality) with importance scores ranging from 0.12 to 0.60. The remaining variables varied in their responses across different algorithms and thus their contribution in governing the species potential distribution varied to a greater extent. For *Rheum webbianum,* bio_15 (Precipitation Seasonality) was the most important contributing variable in determining the species distribution with importance scores ranging from 0.01 to 0.85 followed by bio_14 (Precipitation of Driest Month) and bio_12 (Annual Mean Precipitation) with importance scores ranging from 0.02 to 0.8 and 0.04–0.73, respectively (Table 4).

**Table 4:** Overall and algorithm wise importance scores of the selected bioclimatic variables for *Dactylorhiza hatagirea* and *Rheum webbianum.*

Plant species	Variable	GLM	GBM	GAM	CTA	ANN	SRE	FDA	RF	MP
*Dactylorhiza hatagirea*	bio_01	0.34	0.19	0.60	0.16	0.59	0.35	0.29	0.18	0.49
	bio_02	0.14	0.08	0.49	0.03	0.26	0.27	0.06	0.06	0.12
	bio_03	0.12	0.09	0.60	0.45	0.14	0.06	0.45	0.13	0.19
	bio_08	0.30	0.26	0.70	0.36	0.84	0.30	0.66	0.13	0.36
	bio_12	0.10	0.03	0.45	0.08	0.49	0.18	0.16	0.06	0.14
	bio_14	0.17	0.11	0.42	0.33	0.40	0.15	0.06	0.28	0.36
*Rheum webbianum*	bio_01	0.09	0.03	0.56	0.00	0.34	0.34	0.37	0.03	0.18
	bio_03	0.31	0.11	0.66	0.08	0.15	0.49	0.48	0.18	0.29
	bio_07	0.09	0.04	0.51	0.00	0.25	0.37	0.07	0.04	0.27
	bio_08	0.16	0.07	0.58	0.05	0.38	0.40	0.71	0.06	0.34
	bio_12	0.44	0.18	0.73	0.04	0.40	0.39	0.04	0.06	0.35
	bio_14	0.26	0.10	0.80	0.02	0.19	0.35	0.04	0.10	0.39
	bio_15	0.30	0.35	0.57	0.85	0.43	0.57	0.01	0.10	0.34

### Current distribution

**Figure 2:**
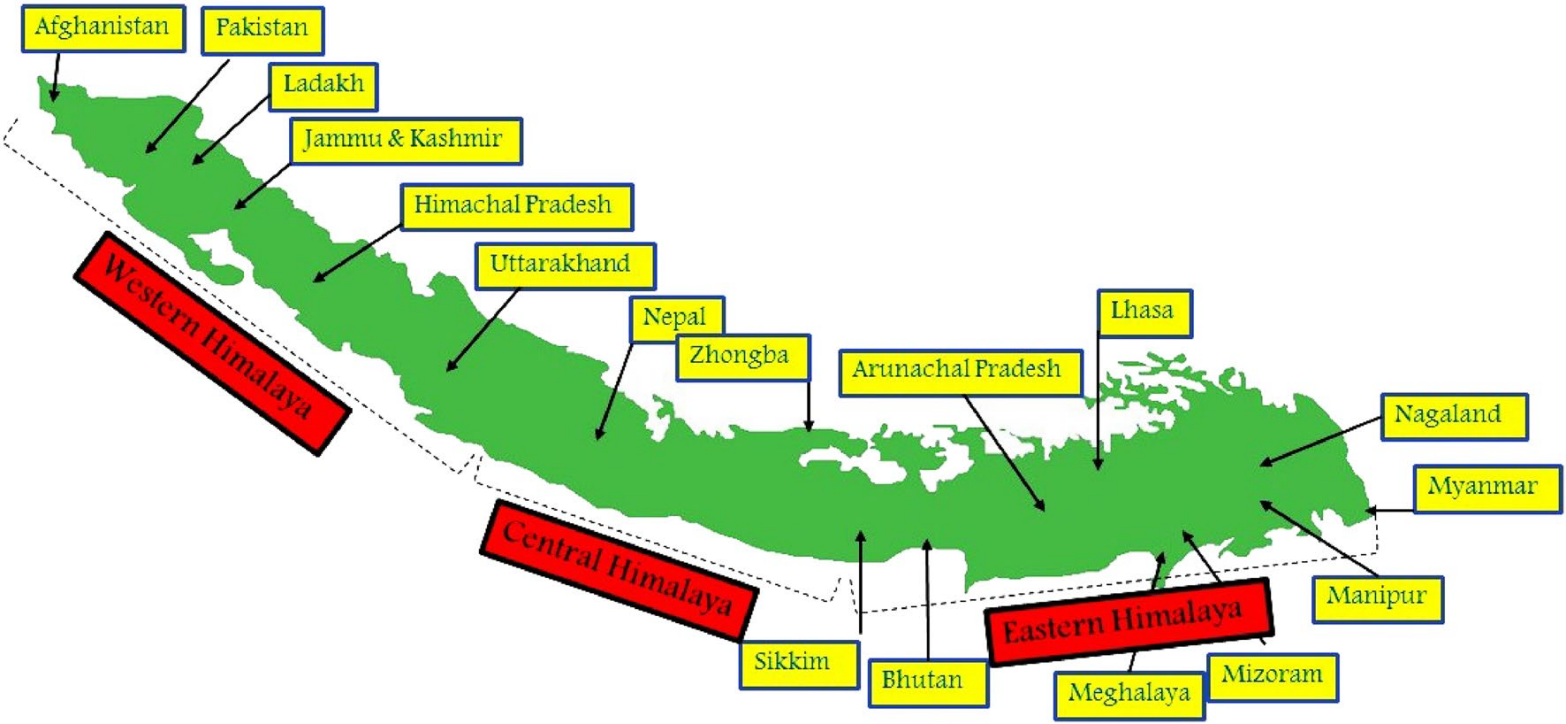
Map of the Himalaya showing the regions of different countries (shape file of the Himalaya extracted in Arc GIS 10.2, Environmental System Research Institute, 2011 https://www.arcgis.com).

**Figure 3:**
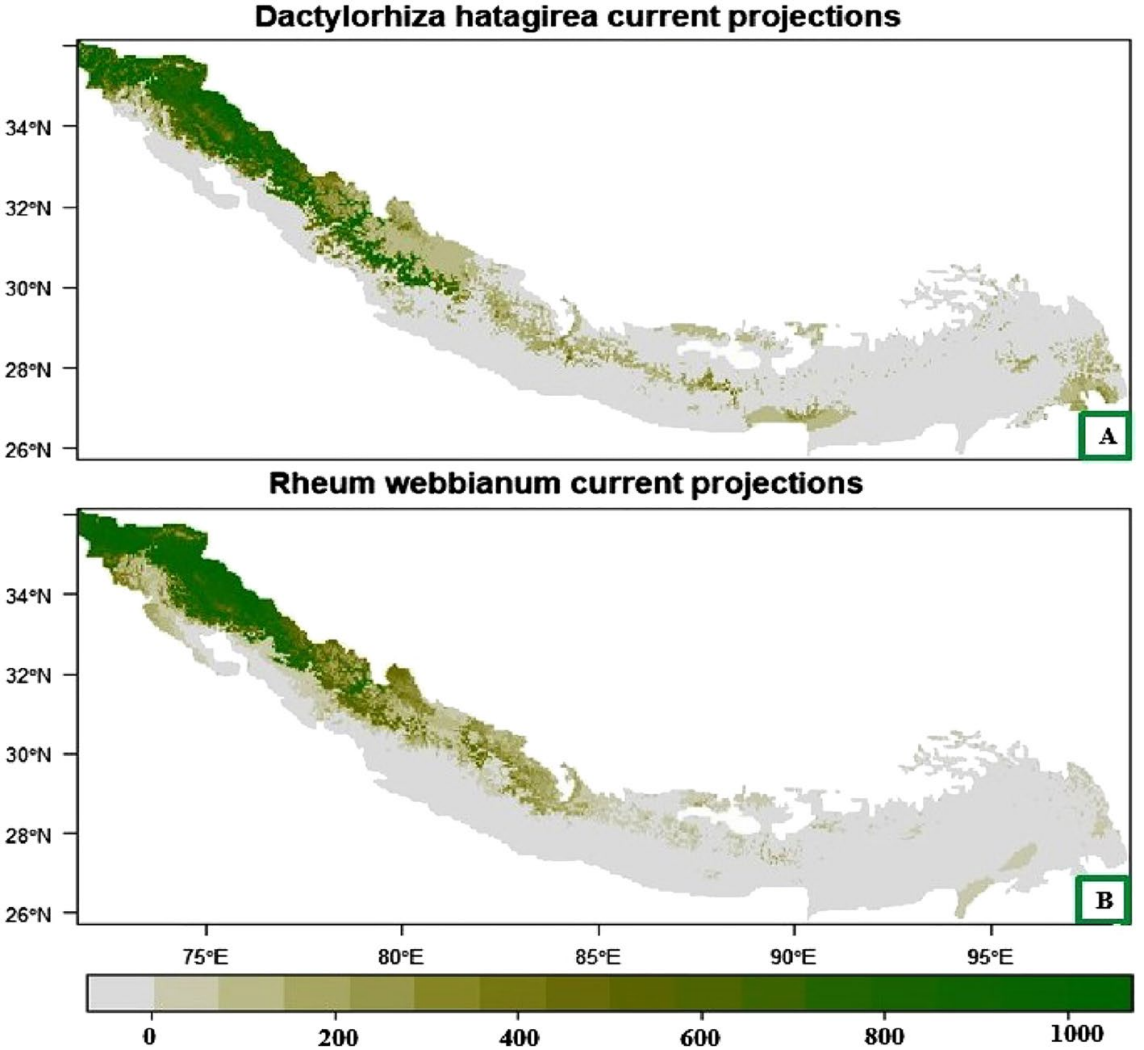
Plot showing the geographic projections of ensemble models for (**A**) *Dactylorhiza hatageria* and (**B**) *Rheum webbianum* under current climatic conditions. The habitat appropriateness class is represented by a scale ranging from 0 to 1000, with 0 denoting the absence of the species, 200–400 represent the habitats with least suitability, 400–600 denoting marginally suitable areas, 600–800 denoting moderately suitable areas, and 800–1000 represent the areas with extremely high habitat suitability.

The Himalayan range extends over to seven Asian countries including India. In India, it is spread over to 11 states and 2 UTs (J&K and Ladakh) occupying an area of (approx.) 2500 km^2^ (Fig. 2). The ensemble model run for the Himalaya showed that under current climatic conditions North-western parts of Jammu and Kashmir and Ladakh UT’s (Ganderbal, Srinagar, Pulwama, Anantnag, Baderwah, Dras, Kargil and Leh), Himachal Pradesh (Hamirpur, Kangra, Bilaspur, Mandi and Solan) and Uttarakhand (Gharwal, Dehradun, Pauri, Haridwar, Bageshwar, Pitthogarh and Almora), northern part of Pakistan bordering Afghanistan (Gilgit, Chitral, Skardu and Muzzafarabad) and north western parts of Nepal (Simikot, Jumla, Mustang, Pokhra, Nawakot, Gurkha, Ramechapp, Kodari, Rasua and Garhi districts) possess highly suitable and optimal climatic conditions for the growth of *Dactylorhiza hatageria*. On the other hand, central part of Nepal (Dharan, Ilam, Amlekghani, Janakpur and Tulsipur) are moderately suitable, while as the central and southern parts of Bhutan (Damphu, Sarpang and Samtse) and Sikkim (Phyaktok, Yathang, Kishong, Chungthan), central and north eastern parts of Arunachal Pradesh (Tezu, Lohit, Namsai, Changlang, Roing and Psighat) show low suitability for *Dactylorhiza hatageria* (Fig. 3A).

For *Rheum webbianum,* western part of Himalayan biodiversity hotspot, covering the north-west areas of India such as Himachal Pradesh (Shimla, Kullu, Kinnaur), Jammu and Kashmir (Rajouri, Poonch, Kishtwar, Ganderbal, Budgam, Baderwah, and Bandipora), central Uttarakhand (Pauri, Chamoli, Tehri, Bagheshwar and Nanital), north Pakistan (Shandur top, Naltar valley, Tarashing, Rupal, Gilgit-Balochistan, Skardu and Astore valley) and north western and central parts of Nepal (Salyan, Silgarhi, Baitad, Gurkha, Baglung and Pyuthan) are highly suitable and optimal climatic conditions for its growth under current climatic conditions, while as northern parts of Uttarakhand and Nepal possess moderate suitability and southern parts of Jammu and Kashmir (Pulwama, Shopian, Anantnag), Himachal Pradesh (Sirmuar and Solan), Uttarakhand (Udham Singh Nagar, Champawat and Nanital) and eastern part of Nepal (Biratnagar and Dhankuta), northern parts of Bhutan (Bumtang and Gasa) and Sikkim (Thangu Valley, Yumthang and Lachung) and southern part of Myanmar (Dawei and Mawlamyine) show low suitability for its growth (Fig. 3B).

### Future potential distribution

**Figure 4:**
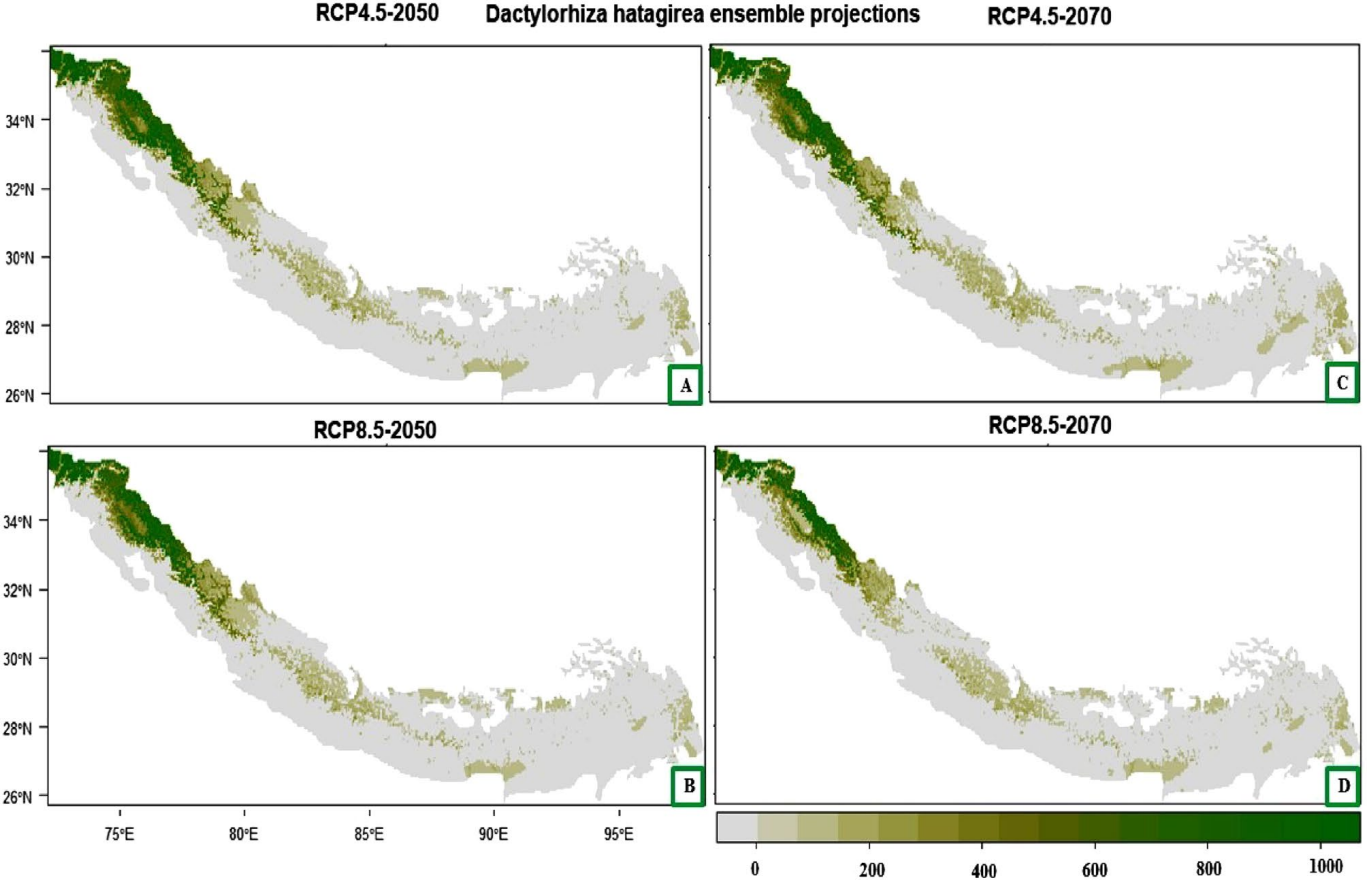
(**A**–**D**) Plot of the predicted habitat suitability for *Dactylorhiza hatageria* under future climate change scenarios.

For *Dactylorhiza hatageria*, there will be a decrease in habitat suitability under all the future climate change scenarios. However, some of the currently suitable areas will consistently remain suitable under future climates also and include most of the western Himalaya (northern Pakistan and northwest regions of India—Jammu and Kashmir, Himachal Pradesh and Uttarakhand), north western, central and south eastern parts of Nepal, and southern parts of Bhutan, Sikkim, Arunachal Pradesh and Myanmar (Fig. 4A–D).

**Figure 5:**
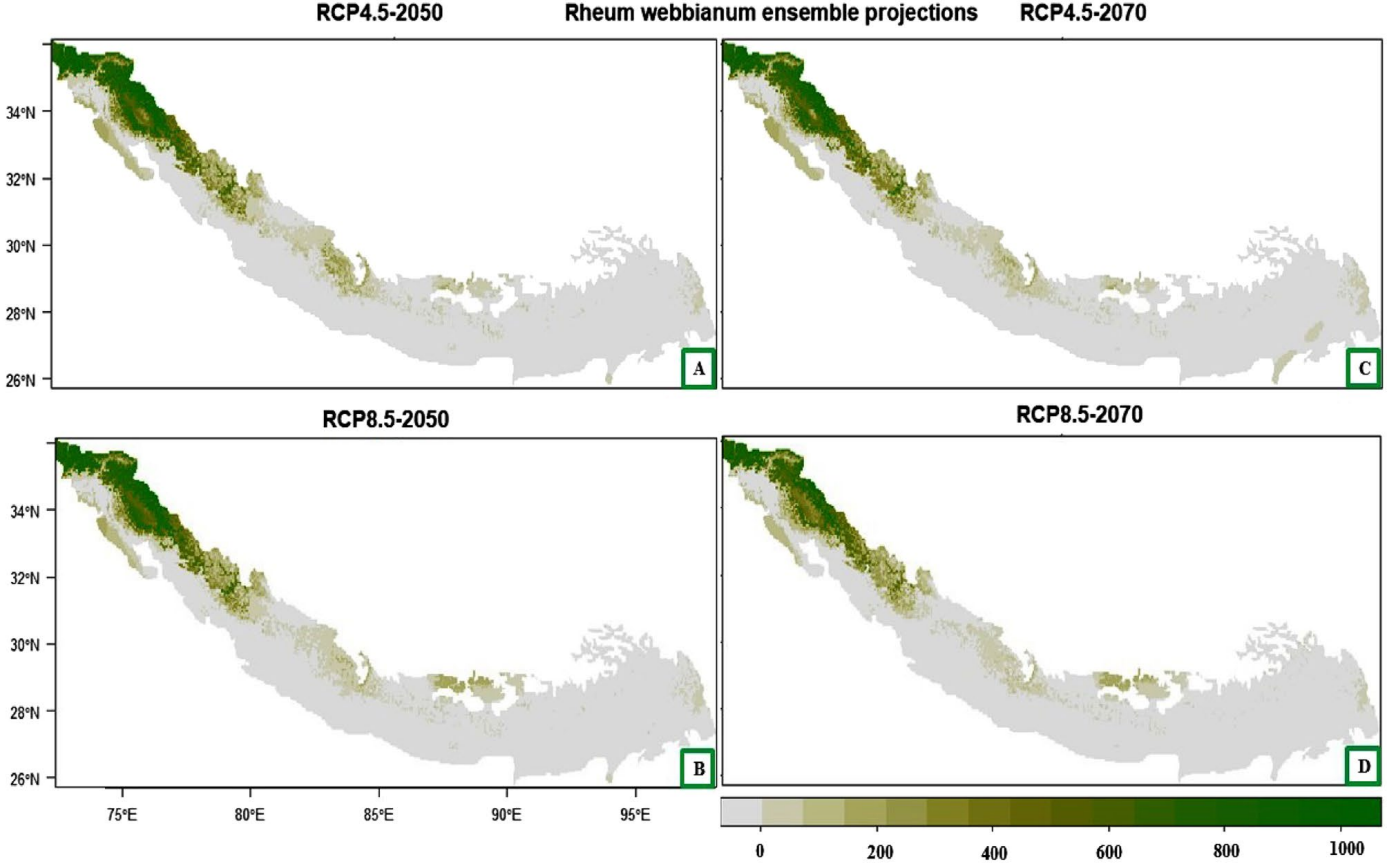
(**A**–**D**) Plot of the predicted habitat suitability for *Rheum webbianum* under future climate change scenarios.

A decrease in the habitat suitability is predicted for *Rheum webbianum* under the future climatic conditions. However, some of the areas that will remain suitable under future climates include most of the western Himalaya (northern Pakistan and northwest regions of India—Jammu and Kashmir, Himachal Pradesh and Uttarakhand) and northern parts of Nepal (Fig. 5A–D).

### Species range change

**Figure 6:**
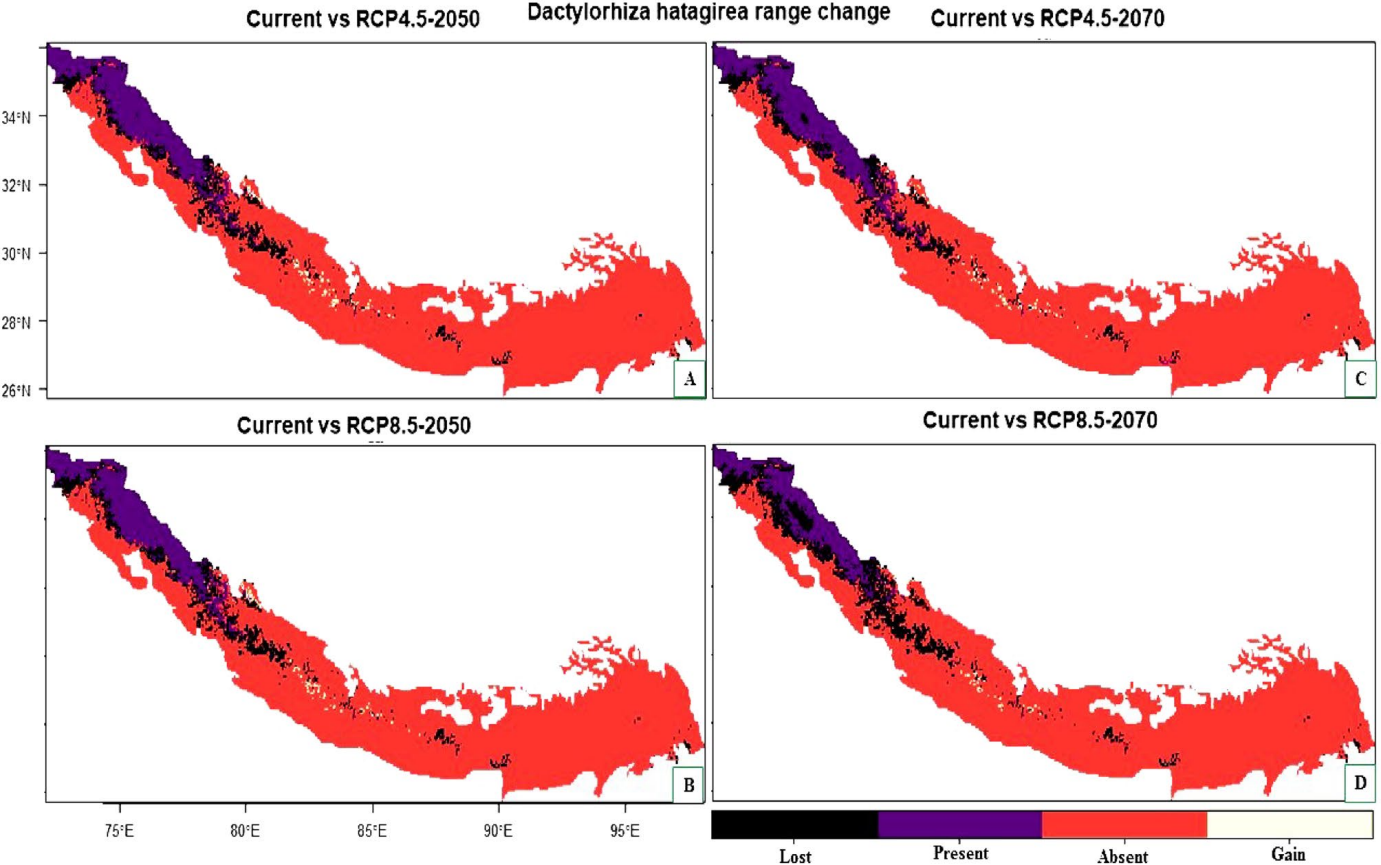
(**A**–**D**) Plot of the predicted range changes for *Dactylorhiza hatageria* between current and future climatic conditions. (**A**) Current VS RCP 4.5 (2050) (**B**) Current VS RCP 8.5 (2050) (**C**) Current VS RCP 4.5 (2070) (**D**) Current VS RCP 8.5 (2070). In scale, black color shows the areas where habitat suitability is predicted to be lost in the future, purple color shows the regions that maintain the habitat suitability in the future climatic scenarios, red color shows the regions where the species is absent while olive color in the scale represents the areas where newly suitable habitats are predicted to appear in the future climate scenarios.

The results of the range change analysis showed that both the studied species will undergo significant range changes under future climatic conditions. This range change will be governed mostly by the loss suitable habitats in the future. More specifically, for *Dactylorhiza hatageria*, the suitable habitat could be reduced by about 28.07% (under RCP4.5 2050), 30.29% (RCP4.5 2070), 31.55% (RCP8.5 2050) and by about 51.41% under RCP8.5 for the year 2070 (Table 5). The areas that are likely to become unsuitable in the future are mostly located towards southern part of Pakistan, central and southern parts of Jammu and Kashmir, Himachal Pradesh and Uttarakhand, northern, western and central parts of Nepal, southern parts of Bhutan and Myanmar (Fig. 6A–D). In contrast, some of the currently unsuitable areas become suitable under future climate with a range expansion of 0.08% (under RCP4.5 2050), 0.36% (RCP4.5 2070), 0.13% (RCP8.5 2050) and 0.07% (under RCP8.5 2070) (Table 5) and include mainly the central part of Nepal (Fig. 6A–D).

**Table 5:** Summary of the range change statistics for *Dactylorhiza hatagirea* under climate change scenarios compared to current climatic conditions.

Scenario	Loss	Absent	Stable	Gain	Loss (%)	Gain (%)	Range change (%)
RCP4.5 2050	41,048	794,652	105,204	125	28.07	0.08	− 27.98
RCP4.5 2070	44,294	794,255	101,958	522	30.29	0.36	− 29.93
RCP8.5 2050	46,147	794,588	100,105	189	31.55	0.13	− 31.42
RCP8.5 2070	75,190	794,676	71,062	101	51.41	0.07	− 51.34

**Figure 7:**
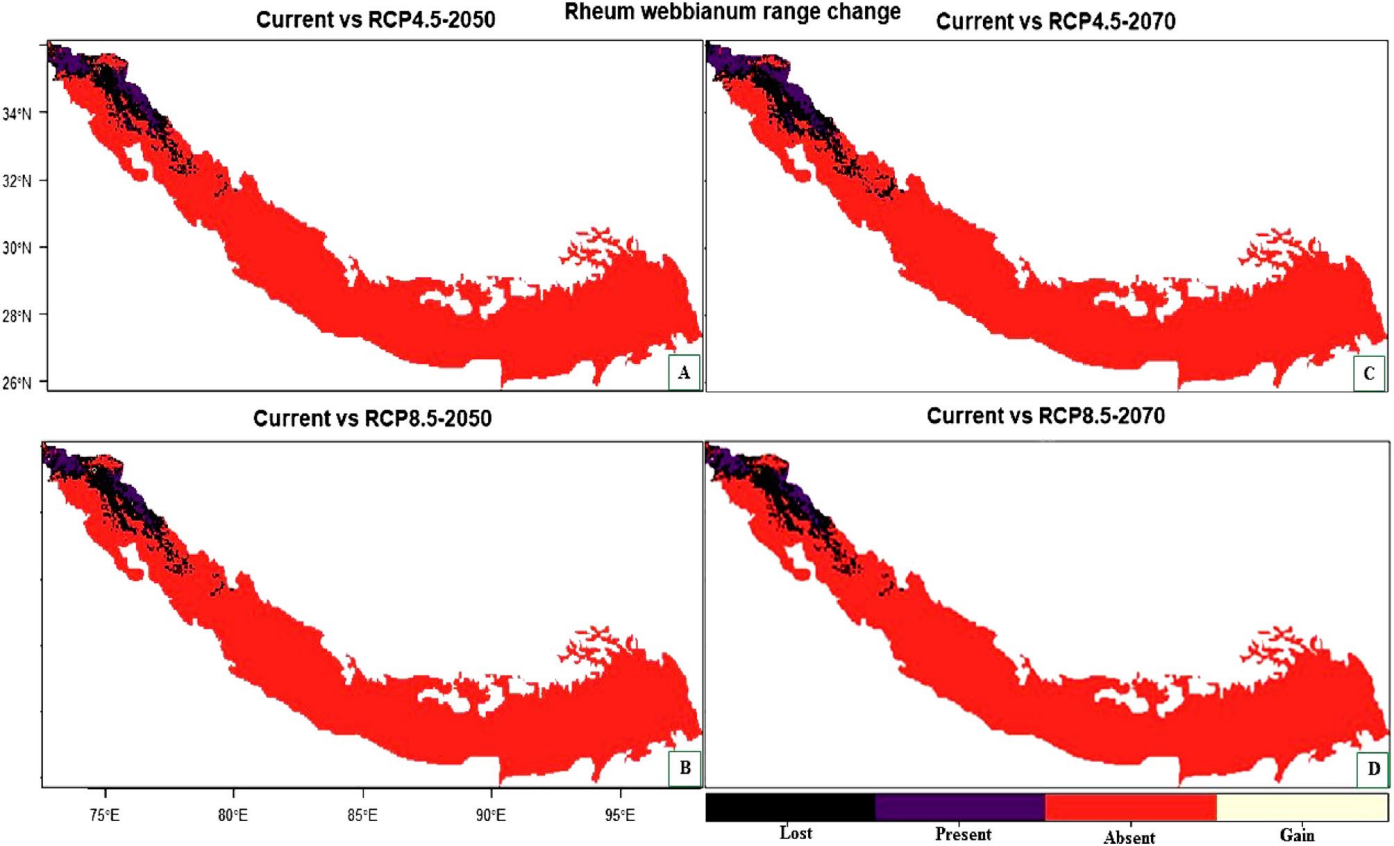
(**A**–**D**) Plot of the predicted range changes for *Rheum webbianum* between current and future climatic conditions. (**A**) Current VS RCP 4.5 (2050) (**B**) Current VS RCP 8.5 (2050) (**C**) Current VS RCP 4.5 (2070) (**D**) Current VS RCP 8.5 (2070).

For *Rheum webbianum*, the suitable habitat could be reduced by about 44.39% (under RCP4.5 2050), 53.49% (RCP4.5 2070), 48.50% (RCP8.5 2050) and by about 70.57% under RCP8.5 for the year 2070 (Table 6). The areas which are likely to become unsuitable for this species in the future include major parts of northern Pakistan, Jammu and Kashmir, Himachal Pradesh and Uttarakhand (Fig. 7A–D). In contrast, some of the currently unsuitable areas will become suitable under future climate with a range expansion of Himalaya 0.004% (under RCP4.5 2050), 0.02% (RCP4.5 2070), 0.006% (RCP8.5 2050) including mainly the western parts of Pakistan, Eastern Himalaya of India and central Nepal (Fig. 7A–D).

**Table 6:** Summary of the range change statistics for *Rheum webbianum* under climate change scenarios compared to current climatic conditions.

Scenario	Loss	Absent	Stable	Gain	Loss (%)	Gain (%)	Range change (%)
RCP4.5 2050	35,893	860,172	44,961	3	44.39	0.004	− 44.39
RCP4.5 2070	43,249	860,157	37,605	18	53.49	0.022	− 53.47
RCP8.5 2050	39,213	860,170	41,641	5	48.5	0.006	− 48.49
RCP8.5 2070	57,059	860,175	23,795	0	70.57	0	− 70.57

### Niche overlap

**Figure 8:**
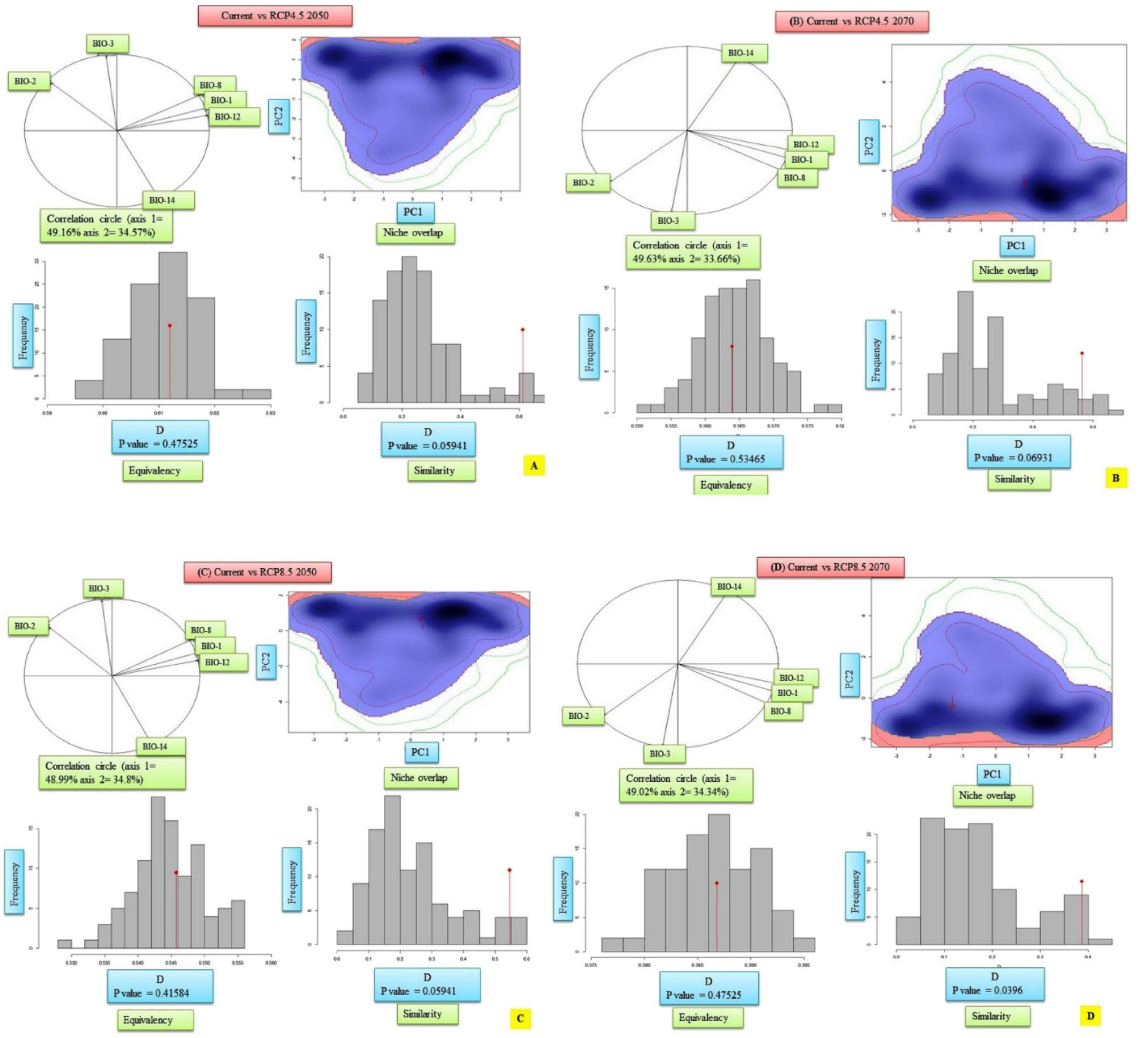
(**A**–**D**) Current and future climatic niches of *Dactylorhiza hatageria*. The correlation circle represents contribution of bioclimatic variables to the first two components main of the PCA-env analyses and the percentage of variation explained by each axis. The blue and red shadings represent density of species occurrence under current and future climatic scenarios. The bar plots represent the results of niche equivalency and similarity tests. The red arrow represents the shift in the climatic niche between current and future climatic conditions.

The results of the niche comparisons between the current and future climatic scenarios of *Dactylorhiza hatageria* revealed that there was moderate level of niche overlap for all the pairs and ranged from 39% (Schoener’s D = 0.39) for current vs. RCP8.5 2070 to 61% (Schoener’s D = 0.61) for current vs. RCP4.5 2050 (Fig. 8A–D; Table 7). The principal component analysis revealed that the variation retained by principal component 1 (PC1) ranged from a minimum of 48.99% in case of current vs RCP 8.5-2050 comparison to a maximum of 49.63% for current vs RCP4.5 2070 comparison (Fig. 8A–D; Table 7). Similarly, for principal component 2 (PC2), the variation retained ranged from a minimum of 33.66% for current vs RCP4.5 2070 comparison to a maximum of 34.80% for current vs RCP8.5 2050 (Fig. 8A–D; Table 7). Further, for each of the pair wise comparisons between the species climatic niche under current and future climatic scenarios, the null hypotheses for niche equivalency were not rejected in any of the pairwise comparison (P > 0.05). In contrast, the niche similarity test for the null hypothesis was rejected only in case of current vs RCP8.5 2070 comparison (Fig. 8A–D; Table 7).

**Table 7:** Niche comparisons and first two principal components between current and future projected distribution of *Dactylorhiza hatagirea*.

Pair	PC1 (%)	PC2 (%)	Overlap (D)	Equivalency test (p value)	Similarity test (p value)
Current vs RCP4.5 2050	49.16	34.57	0.61	0.47525	0.05941
Current vs RCP4.5 2070	49.63	33.66	0.56	0.53465	0.06931
Current vs RCP8.5 2050	48.99	34.8	0.55	0.41584	0.05941
Current vs RCP8.5 2070	49.02	34.34	0.39	0.47525	0.0396

**Figure 9:**
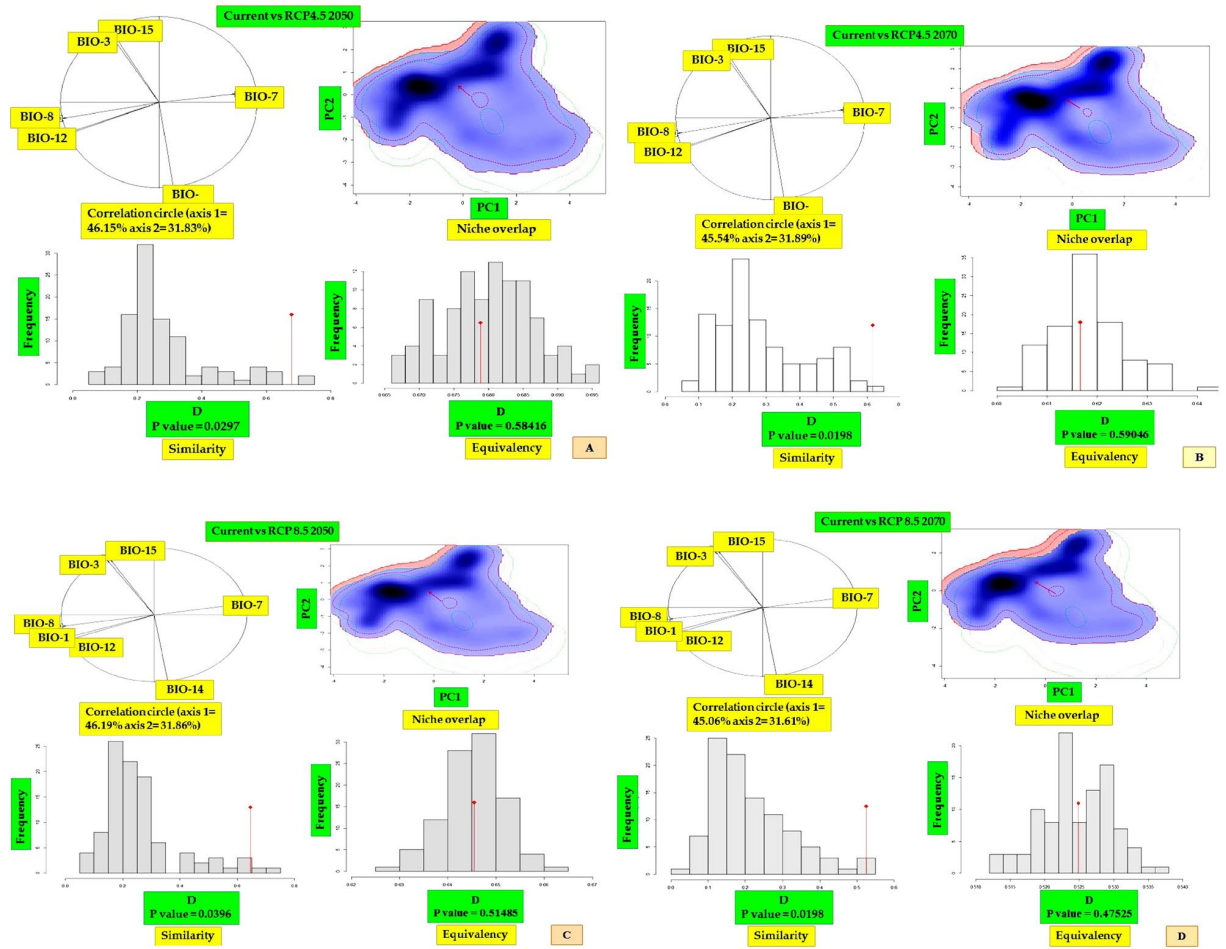
(**A**–**D**) Current and future climatic niches of *Rheum webbianum*. The correlation circle represents contribution of bioclimatic variables to the first two components main of the PCA-env analyses and the percentage of variation explained by each axis. The blue and red shadings represent density of species occurrence under current and future climatic scenarios. The bar plots represent niche the results of niche equivalency and similarity tests. The red arrow represents the shift in the climatic niche between current and future climatic conditions.

For *Rheum webbianum*, the niche comparisons between the current and future climatic scenarios once again showed moderate degree of niche overlap for all the pairs and ranged from 52% (Schoener’s D = 0.52) for current vs. RCP8.5 2070 to 68% (Schoener’s D = 0.68) for current vs. RCP4.5 2050 (Fig. 9A–D; Table 8).The principal component analysis revealed that the variation retained by principal component 1 (PC1) ranged from a minimum of 45.54% in case of current vs RCP4.5 2070 comparison to a maximum of 46.19% for current vs RCP8.5 2050 comparison (Fig. 9A–D; Table 8). Similarly, for principal component 2 (PC2), the variation retained ranged from a minimum of 31.61% for current vs RCP8.5 2070 comparison to a maximum of 31.89% for current vs RCP4.5 2070 (Fig. 9A–D; Table 8). Once again, for each of the pair wise comparisons between the species climatic niche under current and future climatic scenarios, the null hypothesis for niche equivalency was not rejected in any of the pairwise comparison (P > 0.05). On the other side, the niche similarity test for the null hypothesis was rejected in all the cases (P < 0.05) (Fig. 9A–D; Table 8).

**Table 8:** Niche comparisons and first two principal components between current and future projected distribution of the studied species (*Rheum webbianum*).

Pair	PC1 (%)	PC2 (%)	Overlap (D)	Equivalency test (p value)	Similarity test (p value)
Current vs RCP4.5 2050	46.15	31.83	0.68	0.58416	0.0297
Current vs RCP4.5 2070	45.54	31.89	0.62	0.59416	0.0198
Current vs RCP8.5 2050	46.19	31.86	0.65	0.51485	0.0396
Current vs RCP8.5 2070	45.06	31.61	0.52	0.47525	0.0198

## Discussion

Increased global earth temperature and significant alterations in the precipitation pattern^61^ tend to modify the habitat and distribution range of plant species; thereby subjecting them towards greater risk for their extinction^62,63^. Endemic species with narrow distribution range, greater anthropogenic intimidation, smaller population structure and greater habitat specificity could be highly vulnerable to alterations in their distribution range and to degradation or loss of their habitat in near future^64–67^. Forecasting the current and future distribution range through Species distribution modelling (SDMs) is crucial to design different strategic management practices for habitat conservation and management^68,69^. With increasing data availability due to technological advancements in SDMs, ensemble modelling can be a reliable technique^70^. Combining and averaging models using the ensemble approach is thought to reduce model uncertainty and increase its robustness in modelling species distributions accurately^70^. The current work is the upgraded modelling of *D. hatagirea* and *R. webbianum* for current and future distribution by using an ensemble model developed in the biomod2 package in R. Besides, predicting the extent and rate of potential range expansion/contraction and studying the niche dynamics of these species under current and future scenarios is the novelity of this work. The present study area is topographically diverse and thus to cover the heterogeneity of the region upto greater extent, the highest spatial resolution data at 1 km^2^ was used. In mountainous and other areas with steep climate gradients, data at a high (≤ 1 km^2^) spatial resolution are preferred for many applications to capture environmental variation that otherwise can be lost at lower spatial resolutions^71,72^.

The actual niche of the species is generally smaller as compared to the area predicted through model-based predictions, because the climatic variables are not only the sole determinants of habitat suitability^73^. Different biotic and edaphic factors act as limiting variables and provide a subtle role to govern the habitat distribution of a species^74^. It is of utmost significance to point out that when niche modelling is performed for greater geographical areas, climate is usually regarded as the most significant driver of species occurrence^75,76^. Our findings reveal that temperature-based variables (Annual mean temperature, Isothermality, Mean temperature of wettest quarter) gained higher values for predicating the distribution of *D. hatagirea*. Under future climatic scenarios, the plausible explanation for the habitat contraction is that the climate change is expected to replace cold adapted species with warm adapted ones (thermophilization)^10^. Being sub-alpine plant, *D. hatagirea* is highly susceptible to warming and may show migration towards alpine regions as is revealed by slight increase of its habitat suitability in the future. Variable bio_8 (Mean temperature of wettest quarter) corresponds to the juvenile stage of *D. hatagirea* during which majority of the developmental processes occur within the underground perrenating rhizome^77,78^. Under the impact of climate change, increased temperature and earlier melting of snow can lead to earlier, but not necessarily more plant growth due to enzymatic malfunctioning that put constrains in their developmental pathway^79^. Temperature related changes in the phenology affect the dispersal ability of the plants and can thus decrease their habitat suitability in the future^80,81^. Reports have shown that there has been a significant increase in the annual mean temperature and mean temperature of wettest quarter in the western and north western Himalaya^82–84^.

Winter determines the length of the snow-free season as well as the state of soil and plants at the start of the growing season^85^. Plant growth takes place in summer, yet snow cover is equally important in development and morphogenesis of plant communities in alpine and sub-alpine ecosystems^86^. The impacts of warming are reported to be more substantial in winter as compared to other seasons in most parts of the Himalayas^87^. Prediction based estimates reveal that most of the Himalayan glaciers are subjected to loss in volume and mass under the effect of rising temperatures^88^. Consequently, several regions exhibited declining trends in snowfall and retreating glaciers during the recent decades while the higher elevations, experienced increased wintertime precipitation^89^. Precipitation-based variables are predicted to be the most influential variables determining the distribution of *R. webbianum*. Climate change decrease the precipitation of the driest quarter and shows a significant alteration in Mean Annual Precipitation and Mean temperature of wettest quarter^11^. A downward trend in the average annual rainfall in the north-western and western Indian Himalaya was reported by Bhutiyani et al.^87^ and Sontakke et al.^88^. Decrease in the winter precipitation and a rising trend in maximum number of consecutive dry days in winter across the western Indian Himalaya was noted by Dimri and Dash^78^. Ample precipitation in the driest month is an important requirement for the development of *R. webbianum* as it grows in mountain clefts and areas which show less water retention potential^56^. The habitat specificity indicates that *R. webbianum* largely relies on the precipitation-based variables. Non availability of time-to-time precipitation (especially during driest quarter) may put constrains on its habitat suitability.

The results of the current distribution through ensemble model showed that under current climatic conditions western and north western part of Himalayan biodiversity hotspot, possess highly suitable and optimal climatic conditions for the growth of these plant species. The restricted distribution of these plant species towards the western and north western Himalaya can be due to availability of alpine and subalpine regions in these regions with suitable habitats for their growth. These results are supported by the findings of other workers^89–93^ who reported the similar kind of distribution pattern for *Berberis aristata, Trillium govanianum, Picrorhiza kurroa, Lilium polyphyllum* in the northwestern and western regions of the Himalaya. Additionally, the poor seed germination of these plant species outside their natural habitats affect their reproductive ability and consequently lead to their decreased habitat suitability outside their natural abode and restricts their distribution to certain patches only^94–96^.

For the future climate change scenarios, our ensemble model indicated that there occurs a significant contraction in the habitat suitability of these two plant species reaching to its maximum (− 51.34% and − 70.57%) under RCP 8.5 for 2070. The large area from current suitable habitats will become less or not suitable in future and some regions with climatically less or not suitable areas will show higher climatic suitability in the future. The regions that form suitable habitats in future could be used as the conservation sites for rewilding and restoration. The possible reason for their significant niche contraction might be the increased warming in the western Himalaya where the warming occurs at faster rate than predicted for the rest of the world^10,11^.

Under the future climatic projections, the likelihood of these plant species to move and inhabit the areas which lie at greater elevations may reflect the niche shift of the species due to projected increase in earth’s temperature in the future. For RCP 4.5, the emission of the GHGs is low and is predicted to keep most of the regions as suitable habitats, however, under the RCP 8.5 climatic scenarios, more than 50% of the suitable habitats are predicted to be lost for both these species. However, regions of Jammu and Kashmir, Uttarakhand, Northern Pakistan and some regions of Nepal are predicted to remain suitable for *D. hatagirea* and *R. webbianum*. Furthermore, under RCPs 4.5 and 8.5, eastern parts of Uttarakhand, central parts of Nepal and eastern Manipur show a limited range expansion for *D. hatagirea*. For *R. webbianum*, range expansion was predicted in western parts of Pakistan. These results can be related with the predictions for the habitat change of different Himalayan plant species under climate change scenarios^97–101^. Model-based projections of various plant species predicted by different researchers^102–104^ under RCP 8.5 for 2070 also reported significant contraction of potential suitable habitat in response to future climate change scenarios.

Current study is the first attempt to compare the niche dynamics of these species under current and future climatic scenarios. The dynamics of niche change under future climatic circumstances show that there is a loss in niche appropriateness of these plant species, and the suitable habitats will shift to new environmental conditions in the future. This niche dynamics data lend credence to the SDMs' anticipated outcomes, indicating that the current highly appropriate habitat will be constricted in the future and expanding to other which are currently less suited. The evaluation of niche equivalence test clearly shown that the species' environmental niche will not remain precisely the same under current and future climatic scenarios. Similarly, the niche similarity test indicated a considerable degree of overlap between the species under present and future environmental circumstances. Based on these findings, it is clear that species may face comparable but not identical environmental circumstances throughout current and future climate forecasts^105^. There is no reason to reject the null hypothesis of Warren et al.^106^ and the results based on the similarity test allows us to conclude that climatic similarity proposed by Broennimann et al.^107^ will be comparable for these plant species in current and future suitable habitats.

### Implications in conservation

*Dactylorhiza hatagirea* and *Rheum webbianum* are the two prized medicinal herbs of the Himalaya. Incessant over-exploitation of these species has resulted in the progressive dwindling of their natural populations. The unsustained extraction of their underground parts compounded with grazing and trampling has resulted in the degradation of their populations as well as habitats. This loss is further accentuated by the phenomenon of global warming; the impacts of which are believed to be prominent in the mountainous regions. Under such circumstances, an integrated approach involving habitat restoration and identifying the suitable habitats for their reintroduction under climate change scenarios should be a priority. Restoration strategies should be facilitated through rewilding and mass multiplication of these species in and around their natural habitat. Distribution modelling is an efficient method that can provide an early warning system for locating habitats for species under various climatic change scenarios. It provides necessary information to local governments and conservation organisations to choose future suitable sites for the formation of natural habitat reserves. Minimizing the efforts in locating the unknown populations and increasing the accuracy of the field surveys arise to be major breakthrough of the SDM’S. Taking into consideration the threshold and the degree of floristic knowledge of the different regions of the study area, the survey for the new populations of *D. hatagirea* and *R. webbianum* should be prioritized in the North Western, Central and Western parts of different countries that share Himalayan Biodiversity hotspots.

The contraction of the suitable habitats in the future climatic scenarios acts as a signal towards the threat received by these biodiversity heritage relicts. Suitable areas for translocations and reintroduction coincide in many cases with areas that were used in the distribution modelling. We add many more populations to this account and sustainable conservation of these areas will surely arise as a guarantee factor for their persistence. However, the areas that can harbour these plants should be used to manage their populations by reintroduction or translocations. In majority of the cases, the species populations were unprotected and receive greater anthropogenic threat. Offering the protection to the existing natural populations would be an ideal prospective before reintroduction of these species.

Although, SDM cannot substitute fieldworks that are intended towards collection of distributional data, but it can be a valuable ecological tool for data exploration in identifying the potential knowledge gaps in these species. *Dactylorhiza hatagirea* and *Rheum webbianum* show disjunct distribution pattern and application of SDM can direct towards appropriate and more reliable fieldwork design, establishing suitable areas for restoration and identifying potential regions for expansion of natural populations. Inorder to increase the predictive ability of the models in the future, regular update of the occurrence records of newly explored populations needs to be added to the database by the collaborative approach of different government and volunteer groups. In landscape ecology, there is a clear need for such initiatives. These methods will aid in bringing a model closer to authenticity and predicting appropriate locations for rare and endemic species with greater precision.

## Conclusions

In present study, we focused on a BIOMOD ensemble approach to predict the habitat suitability for *Dactylorhiza hatagirea* and *Rheum webbianum*. Suitable habitat distribution was modelled in current and future climate scenarios across their entire distribution range in Himalayan biodiversity hotspots. Temperature based environmental variables show a significant influence on distribution of both these species. Overall, both the species have been documented to be in a state of losing their major part of suitable habitats by year 2050 and 2070 under RCP 8.5. This study provides base line and will be helpful in formulating and implementing the conservation strategies for these species and will enable the conservation biologists to mitigate the climate change effects and human disturbance on their distribution.

## Limitations and future directions

The current predictive distribution modelling study was conducted with the finer resolution of climatic data currently available (i.e., 30 arc sec—approx. 1 km at the equator) considering the fact that the climatic conditions of the Himalaya, vary significantly with shortest distances because of the topographically diverse habitats. Secondly, the near-surface climate is as essential as the aerial climatic factors in determining a species' range^108,109^. However, due to the non-availability of the former for future climatic scenarios, the current study relied heavily on aerial climatic data. The impact of the anthropogenic threats (habitat degradation, overexploitation, change in land use pattern and plant invasion) can all alter a species' range, but these were not taken into consideration in this study. In such a context, the practical application of the current study's findings for restoration initiatives may be limited, unless the intrusion of human driven activities is not managed in the natural habitats of these plant species. In the near future, different research activities should need to incorporate biotic and abiotic elements, as well as dispersion capacities, into SDM projections, resulting in a more robustic and enhanced perspective of species prospective ranges under changing climatic circumstances.

## Materials and methods

### Study area

The “Himalaya” which is considered as a unique repository of medicinal and aromatic plants constitute the study area of current research. Himalaya are distributed across the Asian countries including Afghanistan, Pakistan, India, Nepal, Bhutan and China. More than half of the global population depends directly or indirectly on the Himalaya for their sustenance. These ecosystems being fragile and highly vulnerable to increase in human population, over exploitation of natural resources and impact of climate change have resulted in degradation of these biodiversity hotspots and as such there are more endangered taxa in the Himalaya than anywhere else in the world^110^.

### Target species

*Dactylorhiza hatagirea* is a highly valued medicinal plant of family Orchidaceae. Commonly known as Himalayan Marsh Orchid, it is endemic to Himalaya and show narrow distribution range with specific habitat requirements. It is found inhabiting sub-alpine to alpine regions ranging between the elevations of 2500–5000 m.a.s.l*.* It is native to Asian countries including Afghanistan, Pakistan, India, Nepal, Bhutan and China. The extracts of the plant are used to cure various ailments and is highly valued in both traditional medicinal practices and pharmaceutical sector^96,97^. *Rheum webbianum* is an important medicinal plant of family Polygonaceae. Commonly recognized as Himalayan Rhubarb, it is mainly confined to alpine regions ranging between the elevations of 2400–4300 m.a.s.l. The species is distributed to China, India, Pakistan and Nepal. Roots as well as leaves are medicinally important and find use in both traditional and modern-day systems of medicine^98^.

### Habitat suitability

#### Data collection and evaluation

The distribution data for the two studied species (*Dactylorhiza hatageria* and *Rheum webbianum*) was collected by the authors from intensive field surveys conducted during 2012–2018 and was further supplemented data from the Global Biodiversity Information Facility (GBIF) (http://www.gbif.org/; accessed 29 March 2021) using the *gbif* function available in *dismo* package (https://doi.org/10.15468/d1.xjd7s3 for *D. hatagirea* and https://doi.org/10.15468/d1.mewynz for *R. webbianum*), Botanical Information and Ecology Network (BIEN) (accessed 29 March 2021) and India Biodiversity Portal (IBP) (https://indiabiodiversity.org/; accessed 29 March 2021) databases. The BIEN database was accessed using the BIEN package^60^ in R statistical software version 4.0.3 https://www.r-project.org/). 110 geo-referenced coordinates were located for *D. hatagirea* form Ladakh and Uttarakhand regions while for *R. webbianum*, 80 presence points were recorded from Jammu and Kashmir and Ladakh regions of India with the help of GPS (Mallagien Mobile mapper).

A total of 213 and 198 geo-referenced occurrence records were obtained from the above-mentioned sources as well as filed surveys for *Dactylorhiza hatagirea* and *Rheum webbianum* respectively. These were reduced to 80 and 47 occurrence points after clipping for the study area (i.e. the Himalaya). Each of the occurrence record was thoroughly checked for accuracy before usage, as such records are biased towards geographically suitable and easily accessible areas like cities or areas with higher population density^103^. This results in sampling bias in geographical space. Therefore, in order to eliminate spatial auto-correlation and sample bias, we used spatial thinning, in which the study region was split into 1 × 1 km grid cells and from each cell a single point was chosen randomly. After spatial thinning, a total of 46 and 41 georeferenced points were retained for final dataset in order to model the distribution areas of *Dactylorhiza hatagirea* and *Rheum webbianum* respectively.

#### Environmental data

For modelling the current potential habitat distribution of the selected species across the study region, climatic data was downloaded from (http://www.worldclim.org) WorldClim database, version 1.4^111^. These climatic variables represent maximum, minimum and average monthly, quarterly, and annual precipitation and temperature values measured for 50 years between 1950 and 2000. These environmental variables had a spatial resolution of 30 arc seconds (approx. ~ 1 km resolution at the equator). The bioclimatic variables show a greater degree of correlation among themselves that could affect the performance of model and provide incorrect observations^112^. Therefore, inorder to remove any kind of biases, we performed Pearson’s correlation before modelling, and selected only one variable from each pair of highly correlated variables with correlation coefficient (i.e. r > 0.75). After correlation analysis, a total of six and seven variables were retained for modelling the distribution of *Dactylorhiza hatagirea* and *Rheum webbianum* respectively under current climate conditions (Table 9).

**Table 9:** Bioclimatic variables selected for modelling the distribution of *Dactylorhiza hatagirea* and *Rheum webbianum* in the present study.

*Dactylorhiza hatagirea*	*Rheum webbianum*
BIO-1 (Annual Mean Temperature)	BIO-1 (Annual mean Temperature)
BIO-2 (Mean Diurnal Range)	BIO-3 (Isothermality)
BIO-3 (Isothermality)	BIO-7 (Temperature Annual range)
BIO-8 (Mean Temperature of Wettest Quarter)	BIO-8 (Mean Temperature of Wettest Quarter)
BIO-12 (Annual Mean Precipitation)	BIO-12 (Annual Mean Precipitation)
BIO-14 (Precipitation of Driest Month)	BIO-14 (Precipitation of driest Month)
**–**	BIO-15 (Precipitation Seasonality)

In order to predict the potential future distribution of the species under study, we used the information from the AR5 (fifth assessment report) of IPCC (Intergovernmental Panel for Change Change). Hadley Global Environment Model 2-Earth System (HADGEM2-ES) that represents the simulations for two representative concentration pathways (RCP4.5 and RCP8.5) for the two time periods (i.e. 2050 and 2070) were used. The set of different climatic variables that were used to model the current distribution were also used for predicting the future distributions^36^.

#### Modelling technique

In the present study, we used biomod2 package^22^ within the R statistical software (v 4.0.3; R Development Core Team 2021) to perform the species distribution modelling. Nine different algorithms were used and implemented in biomod2 package, including: Generalised Linear Model (GLM)^113^, Generalised Additive Models (GAM)^114^, Generalised Boosted Models (GBM)^115^, Classification Tree Analysis (CTA)^116^, Flexible Discriminant Analysis (FDA)^32^, Artificial Neural Networks (ANN)^117^, Maximum Entropy (MAXENT)^45^, Random Forest (RF)^47^, and Surface Response Envelope (SRE)^118^.

As these modelling algorithms require both presence and absence datasets, however it is difficult to obtain the actual absence data. Therefore, we randomly generated 500 pseudo-absences within the study area following^119,120^. Since this process of pseudo-absence generation is a stochastic procedure caused by the random selection of the pseudo-absences, therefore, we repeated the procedure three times to address potential sample bias in the pseudo-absence generation^121^.

#### Model calibration

We calibrated the models with 80% of data (training set) and evaluated on the remaining 20% (validation set). This entire procedure was repeated four times. Thus, we obtained a total of 108 models (3 replicate pseudo-absence datasets × 9 algorithms × 4 replicates) for each climatic scenario and time period combination. We evaluated the model performance by repeated data-splitting procedure (cross validation) using two types of evaluation metrics; (1) area under the curve (AUC) of receiver operating characteristics (ROC) and (2) true skills statistics (TSS)^67^. AUC values range from 0 to 1. AUC value between 0.5 and 0.7 indicates poor model run, 0.7–0.9 indicates good and > 0.9 indicated high performance^68^. For True Skill Statistics (TSS) value range from − 1 to + 1. Range below 0.40 indicates poor model run while the values between 0.40 and 0.75 indicates good model performance. The values greater than 0.75 specifies the best model performance^69,122–124^.

#### Model validation

The final ensemble model was built from the individual modelling outputs, for each climatic scenario and time period combination using both committee averaging and weighted-mean approach separately^123^. We only kept models with a TSS score greater than or equal to 0.8 to build the final ensemble models. Thus, we obtained a total of five ensemble projections which correspond to current climatic suitability and four future predicated habitat suitabilities representing two representative concentration pathways (RCP 4.5 and 8.5) for the two time periods (2050 and 2070).

#### Variable importance

For evaluating the relative importance of each climatic variable in governing the distribution of selected plant species, we used permutation procedure^125^. In this procedure we made predictions from a given algorithm after varying only one target variable, while the rest of the variables are kept constant. The variable significance estimate is calculated as 1-correalation score between the original prediction and the prediction made with a permuted variable. Greater values denote a greater importance of the predictor variable whereas a value of 0 means no importance of the variable on the model.

#### Species range change

For each of the selected plant species, we used the BIOMOD *(Range Size)* function in biomod2 package in order to quantify and represent the range change over future climatic scenarios. This function produces two outputs: a table containing summary statistics of species range change and a spatial map that summarizes where species will gain or lose suitable conditions. More specifically, from both the output types, we can get information about for absolute metrics namely “Loss” which represents the number of pixels predicted to be lost by the studied species under climate change; “Absent” representing the number of pixels currently not occupied by the studied species and also not predicted to be suitable under a particular climatic scenario; “Stable” denotes the number of pixels currently occupied by the studied species and also predicted to remain occupied into the future, “Gain” represents the number of pixels which are currently not occupied by the studied species but predicted to be occupied into the future. Finally, from these four metrics, three additional relative metrics were obtained that include “Percentage loss” which corresponds to the percentage of currently occupied sites to be lost and calculated as (Loss/(Loss + Stable); “Percentage gain” corresponding to the percentage of new sites considering the species' current distribution size and calculated as (Gain/(Loss + Stable) and “Range change” which represents the overall projection outcome and is equal to percentage gain − percentage loss.

### Niche overlap

For determining the niche overlap of the plant species under current and future climatic scenarios, modified principal component analysis (PCA-env) was used^126^. Environmental variables are changed into two-dimensional space defined by two principal components. The two-dimensional environmental space is then projected onto a grid cells with a diameter of 100 × 100 and bounded by minimum and maximum PCA values in the background. Smooth key density function was used to overcome sampling bias due to lower number of occurrence data points^127^. Schoener’s D metric was used to determine the extent of niche overlap. It varies from 0 representing no overlap to 1 which represents complete overlap. In order to understand the importance of niche overlap in the geographic area, niche equivalency and similarity tests were performed^126^. Niche equivalence test was performed by the comparison of niche overlap (D) values for current and future climatic scenarios and comparing it to the overlap of null distribution. If the overlap values are significantly lower than niche values, then the null hypothesis of niche equivalency is rejected^126^. A niche similarity test, which assesses whether the niches of two entities being compared are more similar (or different) than would be expected by chance and takes into account the surrounding environmental conditions of the background space across the study region also^128^. We performed the niche analysis using the “ecospat” package in R software^54^.

## Data Availability

All data generated or analysed during this study are included in this published article.
